# *Notes from the Field:* Widespread Transmission of Circulating Vaccine-Derived Poliovirus Identified by Environmental Surveillance and Immunization Response — Horn of Africa, 2017–2018

**DOI:** 10.15585/mmwr.mm6728a6

**Published:** 2018-07-20

**Authors:** Victor A. Eboh, Jeevan K. Makam, Rohit A. Chitale, Chukwuma Mbaeyi, Jaume Jorba, Derek Ehrhardt, Elias Durry, Tracie Gardner, Kamil Mohamed, Christopher Kamugisha, Peter Borus, Eltayeb Ahmed Elsayed

**Affiliations:** ^1^Global Immunization Division, Center for Global Health, CDC; ^2^Division of Viral Diseases, National Center for Immunization and Respiratory Diseases, CDC; ^3^World Health Organization, Geneva, Switzerland; ^4^Office for the Eastern Mediterranean Region, World Health Organization, Amman, Jordan; ^5^Horn of Africa Coordination Office, World Health Organization, Nairobi, Kenya; ^6^Kenya Country Office, World Health Organization, Nairobi, Kenya; ^7^Liaison Office for Somalia, World Health Organization Nairobi, Kenya.

After the declaration of eradication of wild poliovirus type 2 in 2015, all countries using oral poliovirus vaccine (OPV) switched from using trivalent OPV (tOPV) (containing vaccine virus types 1, 2, and 3) to bivalent OPV (bOPV) (containing types 1 and 3) in April 2016 (*1*). Vaccine-derived polioviruses (VDPVs), strains that have diverged from the live vaccine virus during prolonged circulation, can emerge rarely in areas with inadequate OPV coverage and can cause outbreaks of paralysis. Before the global switch from tOPV to bOPV, many circulating VDPV (cVDPV) outbreaks identified globally were caused by type 2 cVDPV (cVDPV2). After the switch, two large cVDPV2 outbreaks occurred in 2017 in the Democratic Republic of the Congo (continuing in 2018) and Syria (*2*,*3*).

Somalia, Kenya, and Ethiopia make up much of the Horn of Africa. Performance indicators for acute flaccid paralysis (AFP) surveillance, an indicator of the sensitivity of surveillance to detect a case of polio, indicate some subnational gaps in these countries, including in areas of Somalia that are inaccessible for polio vaccination activities (*4*,*5*). Sixteen environmental poliovirus surveillance (sewage sampling) sites have been established in these countries to supplement AFP surveillance (Figure).

**FIGURE:**
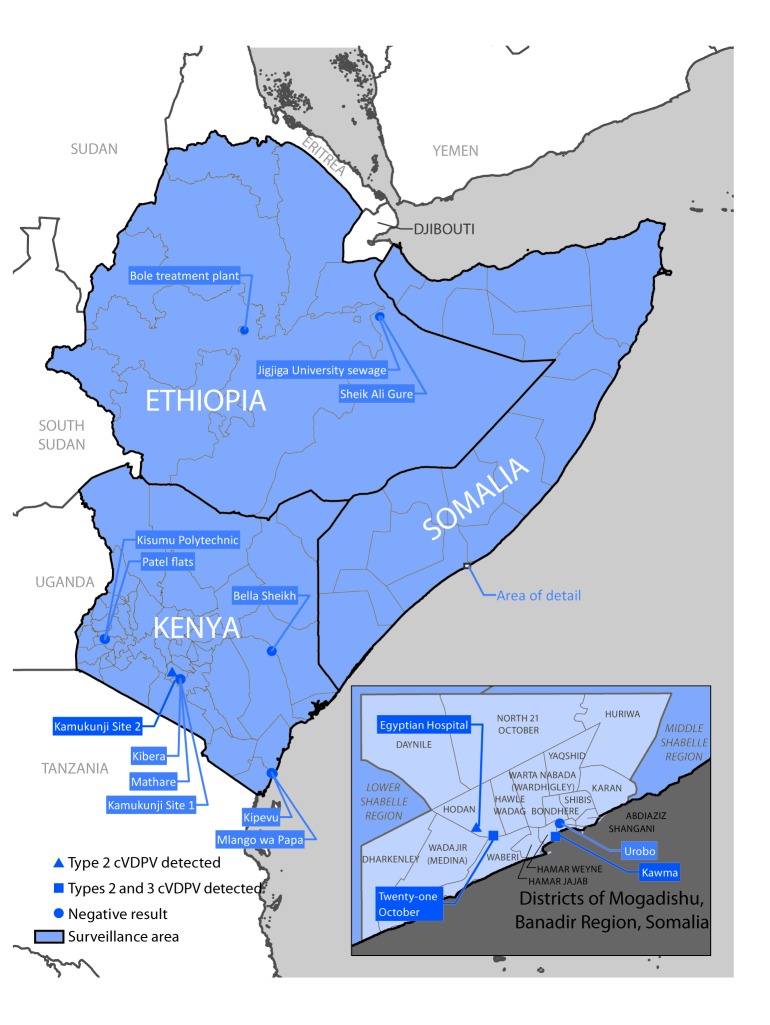
Environmental surveillance for detection of polioviruses — three countries, Horn of Africa region,* 2017–2018 **Abbreviation:** cVDPV = circulating vaccine-derived poliovirus. * Ethiopia, Kenya, and Somalia. Borders for states (Ethiopia), counties (Kenya), and regions (Somalia) are indicated by lines within countries.

Decades of civil unrest and protracted conflict in Somalia have weakened the country’s governance and health care infrastructure, and routine vaccination coverage is estimated to be <50%. Several areas in Somalia are controlled by antigovernment elements that ban vaccination services, leaving approximately 500,000 children aged <5 years unvaccinated (*4*,*5*). Approximately 2 million Somalis are internally displaced or living as refugees in neighboring countries; immunization services might not be effectively extended to a large proportion of displaced and refugee children.

 In October 2017, a VDPV2 isolate was detected from a sewage sample collected from one of four environmental surveillance sites in Banadir, Somalia. The isolate differed from the parental Sabin 2 strain by 38 nucleotides in the VP1 coding region, indicating undetected circulation for >3 years. Genetically related VDPV2 isolates were detected in November 2017 in a sewage sample from the same site. Subsequent detection of genetically related VDPV2 isolates from sewage samples collected in January 2018 from a second site in Banadir confirmed cVDPV2 transmission.

Two response vaccination campaigns using monovalent OPV type 2 (mOPV2) were conducted in December 2017 and January 2018, targeting children aged <5 years in Banadir and the neighboring provinces of Middle Shabelle and Lower Shabelle (Figure); no vaccination was possible in inaccessible areas within these provinces. After detection of a genetically related VDPV2 isolate from a sewage sample collected in February 2018 from a third site in Banadir, a third mOPV2 vaccination campaign was conducted in these same provinces in early May 2018. VDPV2 has not been detected from the fourth sampling site in Banadir.

In March 2018, a VDPV2 isolate differing by 47 nucleotides from the parental Sabin 2 strain was detected in a sewage sample collected in Kamukunji, Nairobi County, in neighboring Kenya. The isolate was genetically linked to the cVDVP2 isolates detected in Banadir (18–25 nucleotide changes), indicating independent circulation of the Banadir and Nairobi VDPV2 lineages for >1 year. VDPV2 has not been detected from samples collected from the other three environmental surveillance sites in Nairobi or from sites in other cities in Kenya, although sample collection was irregular. VDPV2 has not been detected from any of the three environmental surveillance sites in Ethiopia. A limited mOPV2 response vaccination campaign was conducted in Nairobi in May 2018, and two synchronized mOPV2 rounds are scheduled for July and August 2018 in southern and central Somalia, eastern Kenya (including Nairobi), and eastern Ethiopia.

In April 2018, cVDPV type 3 (cVDPV3) isolates (15–17 nucleotide differences from parental Sabin 3 strain) were detected in environmental samples from two sites in Banadir province. In May 2018, cVDPV3 related to the April sewage isolate was identified in stool specimens from two AFP cases in Middle Shabelle province and one AFP case in Hiran province (in which the patient had a coinfection with cVDPV2). A bOPV response vaccination campaign is planned for the southern and central provinces of Somalia. AFP surveillance has been intensified in all three countries through active case finding at health facilities and other reporting sites. This increased surveillance is aimed at closing the gaps in AFP surveillance in all three countries. Further field investigations are ongoing.
